# Marked Increase in Influenza-Associated Acute Myositis Among Children During the 2024–2025 Season: A Two-Season Retrospective Cohort Study From Riyadh, Saudi Arabia

**DOI:** 10.7759/cureus.116385

**Published:** 2026-09-16

**Authors:** Alhommedi Alhabbad, Meshal Albeshr, Basmah AlAtni, Muath AlTuraiki

**Affiliations:** 1 Pediatrics, King Salman Hospital, Riyadh, SAU

**Keywords:** benign acute childhood myositis, child, creatine kinase, emergency department, influenza, influenza a, influenza b, saudi arabia

## Abstract

Background

Influenza-associated acute myositis is well described in children, but most reports count cases without a denominator of infected children. How often influenza is followed by myositis, and whether that frequency shifts between seasons, therefore remains poorly defined. During the 2024-2025 season, pediatricians at our hospital saw an unusual number of children presenting with calf pain and inability to bear weight after laboratory-confirmed influenza. We compared the frequency of acute myositis among influenza-positive children across two matched seasons, and between influenza A and influenza B.

Methodology

We studied all unique children aged 0-14 years with influenza A or B confirmed by rapid antigen testing or polymerase chain reaction who presented to the pediatric emergency department of King Salman Hospital, Riyadh, between 1 November and 28 February of the 2023-2024 and 2024-2025 seasons, regardless of disposition. Each period spanned 120 matched days. Acute myositis required confirmed influenza in the same illness, acute calf or lower-limb pain, gait disturbance or inability to bear weight, creatine kinase above the laboratory upper reference limit of 308 U/L, and no documented alternative explanation. Analyses used risk ratios with 95% confidence intervals, chi-square tests, and a modified Poisson model with robust standard errors.

Results

The cohorts comprised 141 children in 2023-2024 and 173 in 2024-2025. Acute myositis occurred in 19 children (13.5%) in the first season and 73 (42.2%) in the second, an absolute difference of 28.7 percentage points (risk ratio 3.13, 95% CI 1.99-4.93; P<0.001). Pooling both seasons, myositis affected 54 of 107 children with influenza B (50.5%) and 38 of 207 with influenza A (18.4%), with a risk ratio of 2.75 (95% CI 1.95-3.87; P<0.001). The frequency rose within influenza A, from eight of 102 children (7.8%) to 30 of 105 (28.6%), and within influenza B, from 11 of 39 (28.2%) to 43 of 68 (63.2%). Adjusted for influenza type, age, and sex, the season effect persisted (adjusted risk ratio 2.73, 95% CI 1.75-4.26). Case characteristics were similar across seasons: median age 7.7 years in both, peak creatine kinase 4,000 versus 3,710 U/L (P=0.50), and length of stay three versus four days (P=0.49). Myositis-associated admissions rose from 19 to 73 and bed-days from 67 to 264.

Conclusions

Acute myositis was markedly more frequent among influenza-positive children during 2024-2025 than during the matched preceding season. The increase involved both influenza types, with a stronger association for influenza B, while the presentation and biochemical severity of individual cases were unchanged. These findings show an increased local clinical and hospitalisation burden but do not establish increased viral severity, population incidence, or the emergence of a new influenza variant.

## Introduction

Benign acute childhood myositis is a self-limiting complication of viral respiratory infection in school-aged children. It presents with abrupt bilateral calf pain, reluctance or refusal to walk, and raised serum creatine kinase, and resolves over several days [1]. Influenza is the virus most often implicated, and influenza B appears disproportionately often among reported cases in both single-center series and pooled reviews [1-3]. Most of this literature counts myositis cases without a denominator of infected children, so how often influenza is actually followed by myositis, and whether that frequency varies between seasons, remains poorly defined.

The few studies that do use an influenza-positive denominator give very different answers depending on which children the denominator contains. Across three epidemic seasons in an Italian pediatric cohort of 1,046 children aged 0-14 years with laboratory-confirmed influenza, myositis was recorded in 1.4%, and it was strongly associated with influenza B [4]. In a two-center Greek study restricted to children hospitalised with influenza during the 2024-2025 season, 32.7% met criteria for myositis [5]. These figures are not in conflict; they describe different populations, and the denominator has to be stated before any frequency can be interpreted.

During the 2024-2025 influenza season, pediatricians at King Salman Hospital in Riyadh noted an unusual number of children attending with calf pain and inability to bear weight after laboratory-confirmed influenza. Similar impressions circulated informally among pediatricians elsewhere in Saudi Arabia, but informal observation cannot establish whether the frequency changed. Regional data are limited: a Riyadh cohort has described 392 children with benign acute childhood myositis over seven years without an influenza denominator [6], and a single Saudi case report has been published [7].

We therefore compared two matched influenza seasons at one hospital, using all children aged 0-14 years with laboratory-confirmed influenza presenting to the pediatric emergency department as the denominator. The primary objective was to determine whether influenza-associated acute myositis was more frequent during the 2024-2025 season than during 2023-2024. The secondary objectives were to compare the frequency of myositis between influenza A and influenza B, and to compare the two seasons within each influenza type.

## Materials and methods

Study design and setting

This was a single-center retrospective comparative cohort study of two historical seasons, conducted in the pediatric emergency department of King Salman Hospital, Riyadh, Saudi Arabia. The previous season ran from 1 November 2023 through 28 February 2024, and the study season ran from 1 November 2024 through 28 February 2025. Each period contains 120 days; 29 February 2024 was excluded by design so that the two periods were of identical length. Reporting followed the Strengthening the Reporting of Observational Studies in Epidemiology (STROBE) statement for observational studies [8].

Participants and denominator

Eligible participants were all unique children aged 0 through 14 years who presented to the pediatric emergency department during either period with laboratory-confirmed influenza A or influenza B, whether admitted or discharged. The cohort was therefore not restricted to hospitalised children. Influenza testing at this hospital is performed on clinical indication in children with coryzal or influenza-like symptoms or a compatible history, and those who tested positive form the study cohort. The unit of analysis was one unique influenza illness episode; repeat visits or repeat tests belonging to the same illness were consolidated into a single record, and a polymerase chain reaction performed after a rapid test was not counted as a separate episode.

Laboratory confirmation

Influenza was confirmed by a positive rapid influenza antigen test or a positive influenza polymerase chain reaction, and the confirming method was recorded. Where rapid and molecular results for the same illness episode were discordant, the polymerase chain reaction result was used. Viral sequencing, subtyping, and clade analysis were not available at this hospital during either season.

Outcome definition

A child was classified as having confirmed influenza-associated acute myositis only when the record supported all five of the following: laboratory-confirmed influenza A or B during the same illness; acute calf or lower-limb muscle pain; gait disturbance, refusal to walk, or inability to bear weight; a serum creatine kinase concentration above the hospital laboratory upper reference limit of 308 U/L; and no clearly documented alternative explanation for the muscular symptoms. Leg pain without a raised creatine kinase was not sufficient. Refusal or inability to bear weight was recorded as a consequence of pain and was not interpreted as objective neuromuscular weakness unless true weakness had been documented clinically. The identical definition was applied to both seasons.

Consistency of assessment across seasons

Assessment did not change between the two periods. The same group of pediatric emergency physicians staffed the department in both seasons, and no change was made to departmental practice, staffing, or the laboratory service between them. Creatine kinase was measured by the same laboratory assay against the same upper reference limit of 308 U/L throughout. The clinical picture that prompted measurement, calf pain with difficulty walking or refusal to bear weight after a febrile illness, was the same in both years. Measurement followed clinical indication rather than a study protocol in both periods, so a child without muscular symptoms was unlikely to have creatine kinase checked in either season.

Variables and data handling

The following were abstracted for every participant: anonymised study identifier, emergency department visit date, age in years, sex, influenza type, confirming test method, myositis status, and hospital disposition. For children meeting the myositis definition, the presenting muscular features, the peak documented creatine kinase and, for those admitted, the length of stay in days were additionally abstracted. Presenting features were abstracted into predefined categories rather than recorded as free text; each category contained both a lower-limb pain element and a gait or weight-bearing element. The original data sheets were preserved unaltered and all analyses were run against a separate derived dataset. Before analysis, the dataset was checked for duplicate identifiers and episodes, out-of-window visit dates, ineligible ages, missing or invalid categorical values, myositis cases lacking documented symptoms or creatine kinase, implausible creatine kinase or length-of-stay values, and reconciliation of influenza A and B totals against the cohort. No value was imputed and no blank field was converted to a negative response.

Statistical analysis

Categorical variables are reported as counts with the numerator and denominator shown; continuous variables are reported as median with interquartile range. The primary analysis compared the frequency of acute myositis among influenza-positive children between the two seasons using the absolute difference in percentage points and the risk ratio with a 95% confidence interval calculated by the logarithmic method. Secondary analyses compared influenza B with influenza A within each season and in the pooled cohort, and compared the two seasons within each influenza type. Two-sided P values were obtained from the chi-square test without continuity correction; the minimum expected cell count exceeded five in every comparison, so Fisher exact test was not required. Continuous variables were compared between seasons using the Mann-Whitney U test. As a secondary adjusted analysis, a modified Poisson regression with robust standard errors was fitted to all episodes with season, influenza type, age in years, and sex as covariates. Significance was set at a two-sided P<0.05, and effect estimates with confidence intervals are emphasised over P values. Analyses were performed in Python 3.11.15 (Python Software Foundation, Fredericksburg, VA, US) with pandas 3.0.2 (pandas development team, NumFOCUS, Austin, USA), SciPy 1.17.1 (SciPy developers, NumFOCUS, Austin, USA), and statsmodels 0.14.6 (statsmodels development team) libraries, and figures were produced with Matplotlib 3.10.9 (Matplotlib development team, NumFOCUS, Austin, USA).

Ethics

The study was reviewed and approved by the Department of Pediatrics, King Salman Hospital, Riyadh, Saudi Arabia (approval number KSH-PD-23032026), granted on 23 March 2026, before data extraction and analysis began. The requirement for individual informed consent was waived because of the retrospective design, the use of existing clinical records, and the analysis of deidentified data. All records were anonymised before analysis and no identifying information was extracted.

## Results

Cohort

**Figure 1:**
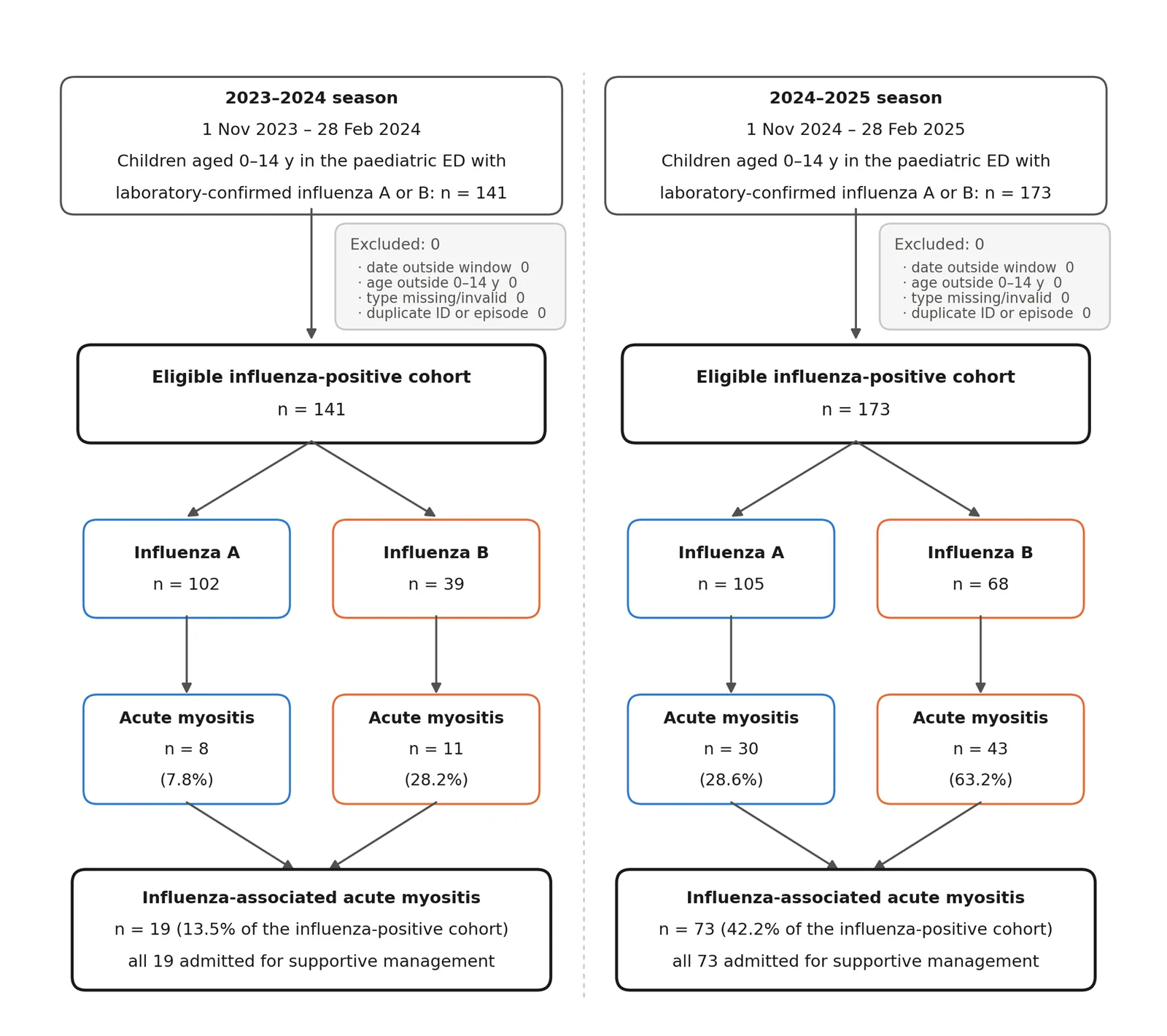
Cohort flow for the two matched influenza seasons Children aged 0-14 years presenting to the pediatric emergency department with laboratory-confirmed influenza A or B during 1 November to 28 February of each season, followed by the numbers excluded at each protocol-defined step, the distribution by influenza type, and the number meeting the acute myositis case definition. Each period spanned 120 days; 29 February 2024 was excluded so that the two periods are identical.

During the 2023-2024 season, 141 unique children aged 0 through 14 years presented to the pediatric emergency department with laboratory-confirmed influenza A or B; during the matched 2024-2025 period, 173 presented. No records were excluded for a visit date outside the season window, an ineligible age, a missing or invalid influenza type, a duplicate identifier, or a duplicate illness episode; thus, the eligible cohorts were 141 and 173, respectively (Figure 1).

No influenza A and B co-infection was recorded in either season.

Of the 141 children in 2023-2024, 89 (63.1%) were male subjects, 102 (72.3%) had influenza A, 39 (27.7%) had influenza B, and influenza was confirmed by rapid antigen testing in 96 (68.1%) and by polymerase chain reaction in 45 (31.9%). Median age was 7.5 years (interquartile range 3.9-10.2, range 0.3-13.8). Of the 173 children in 2024-2025, 113 (65.3%) were male subjects, 105 (60.7%) had influenza A, 68 (39.3%) had influenza B, and influenza was confirmed by rapid antigen testing in 123 (71.1%) and by polymerase chain reaction in 50 (28.9%). Median age was 7.6 years (interquartile range 4.8-10.1, range 0.5-13.8). The two cohorts were therefore similar in age and sex, and differed mainly in the share of infections due to influenza B, which rose by 11.6 percentage points (Table 1).

**Table 1:** Characteristics of the influenza-positive pediatric emergency department cohort, by season IQR: interquartile range; PCR: polymerase chain reaction. Percentages are of the season cohort shown in the column header. Denominators are all unique children aged 0-14 years with laboratory-confirmed influenza A or B presenting to the pediatric emergency department during each 120-day season, regardless of disposition. Hospital disposition was abstracted only for children diagnosed with acute myositis, so the admissions row reports myositis-associated admissions and not total admissions.

Characteristic	2023-2024 season (N=141)	2024-2025 season (N=173)
Age, years, median (IQR)	7.5 (3.9-10.2)	7.6 (4.8-10.1)
Age, years, range	0.3-13.8	0.5-13.8
Male, n (%)	89 (63.1%)	113 (65.3%)
Female, n (%)	52 (36.9%)	60 (34.7%)
Influenza A, n (%)	102 (72.3%)	105 (60.7%)
Influenza B, n (%)	39 (27.7%)	68 (39.3%)
Influenza A and B co-infection, n (%)	0 (0.0%)	0 (0.0%)
Rapid antigen test, n (%)	96 (68.1%)	123 (71.1%)
PCR, n (%)	45 (31.9%)	50 (28.9%)
Acute myositis, n (%)	19 (13.5%)	73 (42.2%)
Myositis-associated admissions, n (%)	19 (13.5%)	73 (42.2%)
Myositis-associated bed-days, n	67	264

Frequency of acute myositis

Acute myositis meeting all five case-definition criteria was identified in 19 of the 141 influenza-positive children in 2023-2024 (13.5%) and in 73 of the 173 in 2024-2025 (42.2%). The absolute difference was 28.7 percentage points and the risk ratio 3.13 (95% CI 1.99-4.93; P<0.001) (Table 2).

**Table 2:** Primary and influenza-type-specific comparisons of acute myositis frequency CI: confidence interval; pp: percentage points. Percentages are of the group denominator shown in the comparison column. Confidence intervals for risk ratios use the logarithmic method. P values are two-sided from the chi-square test without continuity correction; the minimum expected cell count exceeded five in every comparison, so Fisher exact test was not required. Rows labeled Adjusted come from a modified Poisson regression with robust standard errors fitted to all 314 episodes with 92 outcome events, containing season, influenza type, age in years, and sex.

Analysis	Comparison	Group of interest, n (%)	Reference group, n (%)	Difference (pp)	Risk ratio (95% CI)	P
Primary	2024-2025 (N=173) vs 2023-2024 (N=141)	73 (42.2%)	19 (13.5%)	+28.7	3.13 (1.99-4.93)	<0.001
Influenza type	Influenza B (N=39) vs A (N=102), 2023-2024	11 (28.2%)	8 (7.8%)	+20.4	3.60 (1.56-8.27)	0.002
Influenza type	Influenza B (N=68) vs A (N=105), 2024-2025	43 (63.2%)	30 (28.6%)	+34.7	2.21 (1.56-3.15)	<0.001
Influenza type	Influenza B (N=107) vs A (N=207), both seasons	54 (50.5%)	38 (18.4%)	+32.1	2.75 (1.95-3.87)	<0.001
Season within type	Influenza A: 2024-2025 (N=105) vs 2023-2024 (N=102)	30 (28.6%)	8 (7.8%)	+20.7	3.64 (1.75-7.56)	<0.001
Season within type	Influenza B: 2024-2025 (N=68) vs 2023-2024 (N=39)	43 (63.2%)	11 (28.2%)	+35.0	2.24 (1.32-3.82)	<0.001
Adjusted	2024-2025 vs 2023-2024	—	—	—	2.73 (1.75-4.26)	<0.001
Adjusted	Influenza B vs influenza A	—	—	—	2.38 (1.70-3.32)	<0.001
Adjusted	Age, per additional year	—	—	—	1.04 (0.99-1.08)	0.10
Adjusted	Male vs female	—	—	—	1.76 (1.17-2.64)	0.006

In other words, roughly one influenza-positive child in seven met the definition in the earlier season, compared with more than two in five a year later.

Influenza type

In 2023-2024, myositis affected eight of 102 children with influenza A (7.8%) and 11 of 39 with influenza B (28.2%), a risk ratio of 3.60 for influenza B versus influenza A (95% CI 1.56-8.27; P=0.002). In 2024-2025, the corresponding figures were 30 of 105 (28.6%) and 43 of 68 (63.2%), risk ratio 2.21 (95% CI 1.56-3.15; P<0.001). Pooling both seasons, myositis affected 38 of 207 children with influenza A (18.4%) and 54 of 107 with influenza B (50.5%), with a risk ratio of 2.75 (95% CI 1.95-3.87; P<0.001). Influenza B therefore carried the higher frequency in each season and overall.

Change within each influenza type

Within influenza A, the frequency of myositis rose from eight of 102 children (7.8%) to 30 of 105 (28.6%), risk ratio 3.64 (95% CI 1.75-7.56; P<0.001). Within influenza B it rose from 11 of 39 (28.2%) to 43 of 68 (63.2%), risk ratio 2.24 (95% CI 1.32-3.82; P<0.001). Myositis became more frequent in both influenza types between the two seasons. The proportional increase was larger for influenza A (risk ratio 3.64) than for influenza B (risk ratio 2.24). The overall rise therefore cannot be explained by a shift toward influenza B alone.

In the secondary modified Poisson model fitted to all 314 episodes with 92 outcome events, the adjusted risk ratio was 2.73 for the 2024-2025 season versus 2023-2024 (95% CI 1.75-4.26; P<0.001) and 2.38 for influenza B versus influenza A (95% CI 1.70-3.32; P<0.001). Male sex carried an adjusted risk ratio of 1.76 (95% CI 1.17-2.64; P=0.006). Age entered as a linear term was not significantly associated with the outcome (adjusted risk ratio 1.04 per year, 95% CI 0.99-1.08; P=0.10), which reflects the concentration of cases in a narrow middle band rather than any absence of an age effect.

Age and sex distribution of cases

Every one of the 92 children with myositis was between 4.1 and 10.9 years of age. No case occurred below four years, among 63 influenza-positive children in that age range across both seasons, or at 11 years and above, among 49 children. Within the 4- to 10.9-year band the frequency was 19 of 76 children (25.0%) in 2023-2024 and 73 of 126 (57.9%) in 2024-2025. The rise was therefore concentrated in the age group in which this condition is described, rather than spread evenly across the cohort.

Myositis affected 12 of 89 boys (13.5%) and seven of 52 girls (13.5%) in 2023-2024, and 59 of 113 boys (52.2%) and 14 of 60 girls (23.3%) in 2024-2025. The male predominance among cases was therefore present only in the second season, and it accounts for the association with male sex seen in the adjusted model.

Characteristics of the children with myositis

Median age was 7.7 years in both seasons (interquartile range 6.3-9.2 and 5.9-9.4; P=0.86). Male subjects accounted for 12 of the 19 cases (63.2%) in 2023-2024 and 59 of 73 (80.8%) in 2024-2025 (P=0.13). Influenza B was the confirmed type in 11 cases (57.9%) and 43 cases (58.9%) respectively, and diagnosis was by rapid antigen testing in 12 (63.2%) and 46 (63.0%). Bilateral calf pain with calf tenderness and refusal to walk was the most frequently recorded presentation in both seasons, in 14 cases (73.7%) and 36 cases (49.3%).

Median peak creatine kinase was 4000 U/L (interquartile range 2,670-4,190) in 2023-2024 and 3,710 U/L (interquartile range 3,100-4,890) in 2024-2025 (P=0.50), with an overall range of 943 to 14,030 U/L. Every case exceeded the laboratory upper reference limit of 308 U/L; the lowest recorded peak was 943 U/L, or 3.1 times the limit, and the median corresponded to 12.1 times the limit. Every child meeting the definition was admitted, with a median stay of three days (interquartile range 3-4) and four days (interquartile range 3-4) respectively (P=0.49) and an overall range of three to five days. Neither the biochemical severity nor the duration of admission differed between seasons (Table 3).

**Table 3:** Characteristics of the children with influenza-associated acute myositis IQR: interquartile range; PCR: polymerase chain reaction. Percentages are of the myositis cases in each season, as shown in the column header. P values compare the two seasons: Mann-Whitney U test for age, creatine kinase, and length of stay; Fisher exact test for sex and influenza type.

Characteristic	2023-2024 (N=19)	2024-2025 (N=73)	P
Age, years, median (IQR)	7.7 (6.3-9.2)	7.7 (5.9-9.4)	0.86
Age, years, range	4.1-10.9	4.2-10.9	—
Male, n (%)	12 (63.2%)	59 (80.8%)	0.13
Female, n (%)	7 (36.8%)	14 (19.2%)	—
Influenza A, n (%)	8 (42.1%)	30 (41.1%)	1.00
Influenza B, n (%)	11 (57.9%)	43 (58.9%)	—
Rapid antigen test, n (%)	12 (63.2%)	46 (63.0%)	—
PCR, n (%)	7 (36.8%)	27 (37.0%)	—
Bilateral calf pain, calf tenderness, refusal to walk, n (%)	14 (73.7%)	36 (49.3%)	—
Bilateral calf pain, difficulty walking, n (%)	3 (15.8%)	23 (31.5%)	—
Severe bilateral calf pain, calf tenderness, inability to bear weight, gait disturbance, n (%)	2 (10.5%)	14 (19.2%)	—
Peak creatine kinase, U/L, median (IQR)	4,000 (2,670-4,190)	3,710 (3,100-4,890)	0.50
Peak creatine kinase, U/L, range	943-14,030	943-13,470	—
Admitted, n (%)	19 (100.0%)	73 (100.0%)	—
Length of stay, days, median (IQR)	3 (3-4)	4 (3-4)	0.49
Length of stay, days, range	3-5	3-5	—
Total bed-days, n	67	264	—

Monthly distribution and admission burden

**Figure 2:**
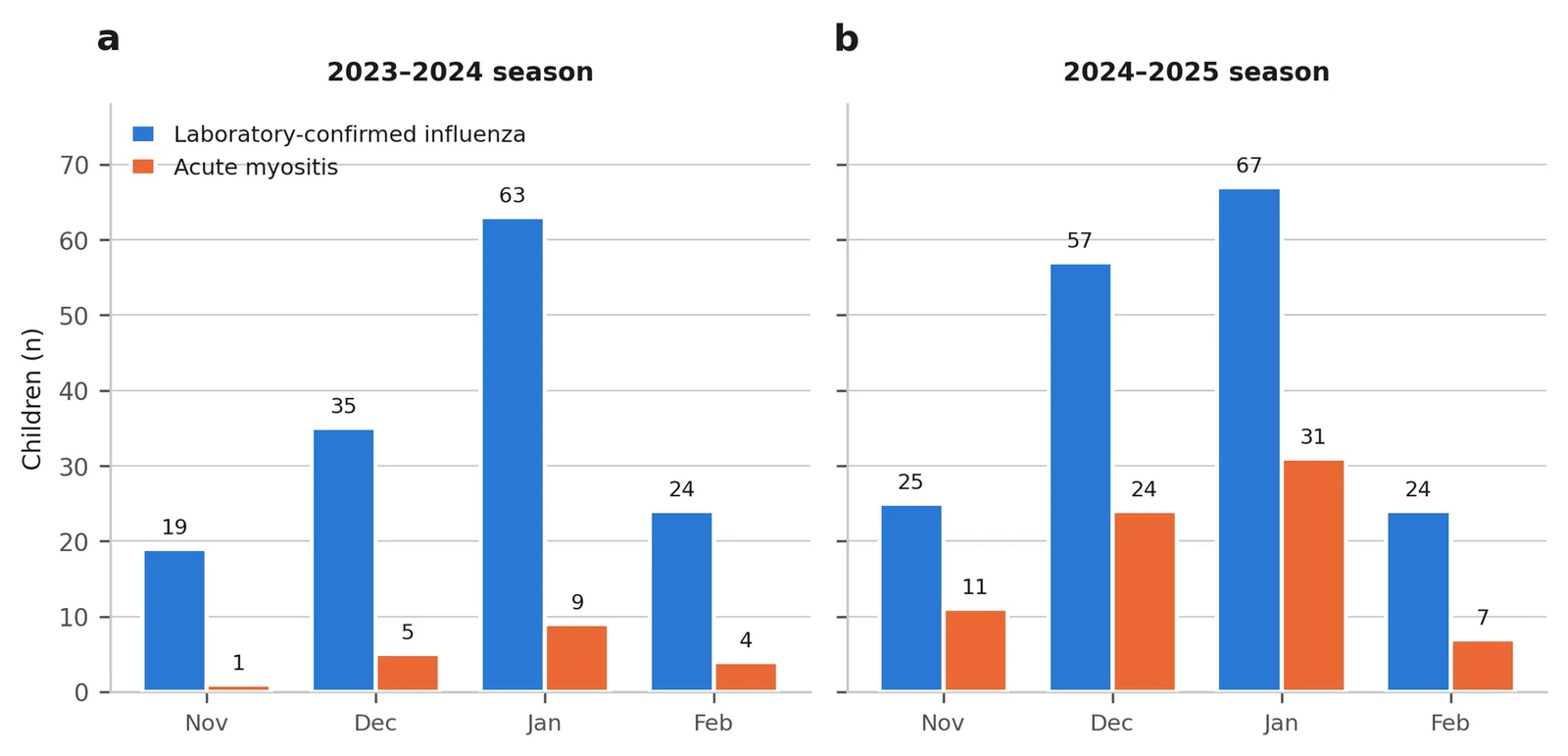
Monthly distribution of laboratory-confirmed influenza and acute myositis across two matched seasons (a) 2023-2024 season. (b) 2024-2025 season. Bars show children aged 0-14 years presenting to the pediatric emergency department during each month of the 1 November to 28 February study period. In every monthly pair the first bar is all children with laboratory-confirmed influenza A or B, and the second is those among them who met the acute myositis case definition. Myositis cases are a subset of the influenza cases for that month rather than a separate group, so the two bars should not be added together. Every child meeting the definition was admitted. Both panels use the same vertical scale.

Confirmed influenza episodes by month were 19, 35, 63, and 24 for November, December, January, and February of 2023-2024, and 25, 57, 67, and 24 for the corresponding months of 2024-2025. Myositis cases followed the same monthly shape in both seasons, at one, five, nine, and four, and then 11, 24, 31, and seven, peaking in January alongside influenza activity (Figure 2).

The myositis curve therefore tracked influenza circulation rather than forming a separate outbreak.

Myositis-associated admissions numbered 19 in 2023-2024 and 73 in 2024-2025, accounting for 67 and 264 hospital bed-days respectively, a near fourfold increase in occupied bed-days across a 120-day window.

## Discussion

Among children presenting to this pediatric emergency department with laboratory-confirmed influenza, acute myositis was three times as frequent during the 2024-2025 season as during the matched preceding season, rising from 13.5% to 42.2% of the influenza-positive cohort. That figure is the frequency of recognised myositis among influenza-positive children attending one emergency department, not the risk of myositis after influenza infection in the community, and the distinction governs how the rest of these findings should be read. The increase was not confined to one influenza type: the frequency more than tripled within influenza A and more than doubled within influenza B, and the season effect persisted after adjustment for influenza type, age, and sex. Influenza B carried the higher absolute frequency in both seasons and in the pooled cohort, consistent with the pattern reported in Italian, Greek, Polish, and Saudi series and in a recent scoping review [1-6].

The frequency we observed was far higher than the 1.4% reported in an Italian cohort that used a comparable influenza-positive denominator of children aged 0-14 years [4]. That difference reflected what the denominator contains rather than a difference in biological risk. Our denominator comprised children who were unwell enough to attend an emergency department, were tested on clinical indication, and tested positive. Children with mild influenza managed without testing never entered it, while children with prominent calf pain and difficulty walking were among the most likely to be tested and to have creatine kinase measured, so the numerator was preferentially ascertained relative to the denominator. Our figure should be read as the frequency of recognised myositis among influenza-positive attendees at one hospital, not as the risk of myositis after influenza infection in the community. The Greek study of the same 2024-2025 season reported 32.7% among children hospitalised with influenza [5], closer to our value, but it rested on a narrower denominator still, and the two proportions should not be equated.

The Riyadh cohort that described 392 children with benign acute childhood myositis over seven years counted cases without an influenza denominator [6], so it cannot show whether the frequency changed in any given season. That is the gap a matched two-season design with a fixed denominator addressed. Because the same case definition, denominator, and 120-day calendar window were applied to both seasons at the same site, the comparison is internally consistent even though the absolute level is site-specific.

Two features of the data suggested that what changed was the number of affected children rather than the nature or severity of the illness. The clinical and biochemical profile of cases was essentially unchanged: median age was 7.7 years in both seasons, and neither peak creatine kinase nor length of stay differed. All cases also fell within the 4- to 11-year band characteristic of benign acute childhood myositis, and the recorded presentations, median creatine kinase, and length of stay are consistent with published series [1,2,6]. Admission in this cohort reflected calf pain, impaired weight bearing, and the need for intravenous hydration and analgesia rather than organ dysfunction or a complicated course.

The proportion of influenza B rose from 27.7% to 39.3% of confirmed infections between the two seasons. Given the stronger association of myositis with influenza B, a shift in circulating type contributed to the overall rise, but it cannot be the whole explanation, because the frequency also more than tripled within influenza A. Why the frequency rose within each type is not answerable from these data. Only rapid antigen testing and polymerase chain reaction were available at this hospital, with no sequencing, subtyping, or clade analysis, so a change in circulating virus can be raised only as a hypothesis for future work. Rising clinical awareness within the same team was the remaining alternative. Staffing, the assay, the reference limit, and the presenting picture that prompted testing were unchanged between the seasons, so the usual mechanisms of differential testing do not apply here, but once several cases have been recognised early in a season clinicians asked about calf pain more readily. That is not testable from retrospective records. Prospective studies with systematic creatine kinase measurement in all influenza-positive children, combined with viral subtyping, would separate these possibilities.

For clinicians, the practical implication is one of local burden and of pattern recognition. Acute myositis deserves consideration in any school-aged child with laboratory-confirmed influenza who develops calf pain or refuses to bear weight, and creatine kinase should be measured when those features are present. Management in this cohort was supportive and recovery was rapid, yet myositis-associated admissions rose from 19 to 73 and consumed 264 rather than 67 bed-days across a 120-day window. Departments serving similar populations may find it useful to anticipate this pattern in seasons where influenza B circulates widely.

Limitations

The main limitation is ascertainment. Influenza testing was performed on clinical indication rather than universally, and creatine kinase was measured when clinically indicated rather than in every influenza-positive child. A child with calf pain and difficulty walking is among the most likely to be tested for influenza and to have creatine kinase checked, so myositis cases are identified more completely than the influenza-positive denominator they sit within. Mild or subclinical myositis was not counted at all.

What did not vary is also relevant. The same physicians staffed the department in both seasons, the assay and its reference limit were unchanged, the presenting picture that prompted testing was the same, and the case definition, denominator, and 120-day calendar window were applied identically at one site. Those constants remove the usual mechanisms by which testing practice differs between periods. They do not exclude the possibility that the same clinicians asked about calf pain more readily once several cases had been recognised early in the 2024-2025 season, and that possibility cannot be tested from retrospective records.

This was a single-center study, and the absolute frequencies reported here apply to children attending one pediatric emergency department in Riyadh. They should not be generalised to the city, the country, or to the risk of myositis after influenza infection in the community. Hospital disposition was abstracted only for children who met the myositis definition, so the proportion of all influenza-related admissions attributable to myositis cannot be estimated. No viral sequencing, subtyping, or clade analysis was available. Data were abstracted retrospectively, with presenting features recorded in predefined categories rather than as free text, and no follow-up beyond discharge was available.

## Conclusions

Influenza-associated acute myositis deserves recognition as a predictable seasonal contributor to pediatric emergency workload rather than as an occasional curiosity. Where influenza circulates widely, and particularly where influenza B predominates, a proportion of school-aged children will arrive unable to walk, and the diagnosis turns on a single question about calf pain and one laboratory test. Recognising the pattern early spares children unnecessary imaging and neurological referral, shortens the path to supportive care, and allows departments to anticipate a run of brief admissions rather than absorbing them as a surprise.

The wider lesson concerns denominators. The frequency of a post-infectious complication carries little meaning unless the population at risk is stated, and proportions drawn from different denominators cannot be set beside one another as though they measured the same quantity. Studies that fix the denominator, hold the case definition constant, and compare like calendar periods are what will eventually show whether the burden of this condition is genuinely changing or whether clinicians are simply looking for it more carefully. Until that work exists, the prudent course is to ask about leg pain in every school-aged child with influenza, to measure creatine kinase when the question arises, and to report any resulting frequency alongside the denominator from which it was drawn. These findings show an increased local clinical and hospitalisation burden. They do not establish increased viral severity, population incidence, or the emergence of a new influenza variant.
